# Correction: A team of heterochromatin factors collaborates with small RNA pathways to combat repetitive elements and germline stress

**DOI:** 10.7554/eLife.32516

**Published:** 2017-10-05

**Authors:** Alicia N McMurchy, Przemyslaw Stempor, Tessa Gaarenstroom, Brian Wysolmerski, Yan Dong, Darya Aussianikava, Alex Appert, Ni Huang, Paulina Kolasinska-Zwierz, Alexandra Sapetschnig, Eric A Miska, Julie Ahringer

McMurchy AN, Stempor P, Gaarenstroom T, Wysolmerski B, Dong Y, Aussianikava D, Appert A, Huang N, Kolasinska-Zwierz P, Sapetschnig A, Miska EA, Ahringer J. 2017. A team of heterochromatin factors collaborates with small RNA pathways to combat repetitive elements and germline stress. *eLife* **6**:e21666. doi: 10.7554/eLife.21666.

Published 15, March 2017

Due to a copy/paste error in the originally published version of Figure 2—source data 1, the peak calls listed for HPL-2 were a duplication of the peak calls for MET-2.

The article has been corrected accordingly.

